# Comparison of Perioperative Analgesic Efficacy of Lidocaine Intratesticular Block and Spermatic Cord Instillation in Feline Castration

**DOI:** 10.3390/vetsci12020111

**Published:** 2025-02-02

**Authors:** Tossawarn Makpunpol, Yanika Chanrobru, Passawan Thanapaisal, Manawee Kuropakaranan, Piyasak Wipoosak, Suvaluk Seesupa, Supranee Jitpean, Duangdaun Kaenkangploo, Preenun Jitasombuti, Chalermkwan Nonthakotr, Nitaya Boonbal, Somphong Hoisang, Wanwisa Chaoum, Naruepon Kampa, Thanikul Srithunyarat

**Affiliations:** 1 Veterinary Teaching Hospital, Faculty of Veterinary Medicine, Khon Kaen University, Khon Kaen 40002, Thailand; tossma@kku.ac.th (T.M.); piyawi@kku.ac.th (P.W.); chalno@kku.ac.th (C.N.); nitabo@kku.ac.th (N.B.); sompho@kku.ac.th (S.H.); 2 Division of Surgery, Faculty of Veterinary Medicine, Khon Kaen University, Khon Kaen 40002, Thailand; yanika.ch@kkumail.com (Y.C.); th.passawan@kkumail.com (P.T.); manaweeku@kkumail.com (M.K.); suvalukse@kku.ac.th (S.S.); supraneeji@kku.ac.th (S.J.); duakae@kku.ac.th (D.K.); preenun@kku.ac.th (P.J.); wanwisa.cha@kkumail.com (W.C.); naruepon@kku.ac.th (N.K.)

**Keywords:** cat, local anesthesia, neutering, spermatic cord, instillation

## Abstract

Intratesticular block is an effective local anesthetic technique for castration; however, its use may be limited in cases of testicular masses or infections. Instillation is a simple technique used in several procedures, though studies investigating its application in feline castration remain scarce. Therefore, this study aimed to compare the perioperative analgesic efficacy of lidocaine intratesticular block and spermatic cord instillation in feline castration. Forty-five male cats were randomly divided into three groups: the intratesticular block group (IT), the spermatic cord instillation group (S), and the control group (C). All cats were anesthetized using the same protocol and both techniques were performed based on group assignments. Lidocaine (2 mg/kg) was administrated according to the designated route, while 0.9% normal saline was used for the alternative route and in the C group. Intraoperative parameters were recorded, showing that noninvasive blood pressure in the IT and S groups was significantly lower than in the C group. Although both lidocaine intratesticular block and spermatic cord instillation effectively reduce surgical noxious stimulation, intratesticular block is recommended as it provides more stable intraoperative vital signs and less postoperative pain. Therefore, spermatic cord instillation can be considered as an optional technique for managing pain in feline castration.

## Simple Summary

Castration is a common surgery that induces mild to moderate pain intensity. Effective pain management, particularly local anesthesia in addition to general anesthesia, is recommended. Intratesticular block is a well-established technique for castration; however, its use may be limited in cats with testicular masses or infections. Instillation is a simple and effective local anesthetic technique used in various surgeries, though studies on its use for castration are limited. This study aimed to compare the analgesic efficacy of intratesticular block and spermatic cord instillation in cats undergoing castration. The results demonstrated that both techniques effectively reduce noxious stimulation during surgery, with no significant differences observed between the two methods. Therefore, the spermatic cord instillation technique can be considered as a local anesthetic option for feline castration.

## 1. Introduction

Castration is one of the most common surgeries performed in cats. The primary benefit of this procedure during the pediatric period is the improvement of behaviors by reducing aggressive and sexual behaviors, including urine spraying [1,2,3]. Although castration causes mild to moderate pain, appropriate pain management is still necessary [4]. The recommended approach for pain management in feline castration includes general anesthesia combined with preventive and multimodal analgesia. General anesthesia and inhalation anesthesia can induce side effects such as bradycardia, hypotension, and hypercapnia [5,6]. Preventive and multimodal analgesia, in addition to general anesthesia, can reduce intraoperative noxious stimulation resulting in minimizing the requirement of anesthetic induction and maintenance during surgery, reducing the potential side effects of general anesthesia, and improving postoperative recovery [5,7,8]. Pain assessment in cats can be performed using recommended clinical metrology instruments, including the Glasgow composite measure pain scale-feline (CMPS-feline), the UNESP-Botucatu multidimensional composite pain scale (UNESP), the Colorado acute pain scale feline (CSU-feline), and the feline grimace scale [7,8]. Adequate perioperative pain management reduces pain scores and analgesia requirements after surgery.

Local anesthesia plays an important role in a multimodal analgesia approach. Lidocaine is the most widely used local anesthetic that has rapid onset of action (less than 5 min) and a short duration effect (approximately 1 h) [9]. However, cats are particularly susceptible to lidocaine toxicity as systemic absorption and intravenous administration may cause serious adverse effects, including central nervous system depression, seizures, and cardiovascular depression [10]. The toxic effects of lidocaine may occur in cats at a dose approaching 6 mg/kg, with seizures being induced at an intravenous dosage of 11.7 ± 4.6 mg/kg. As a precaution, it is advised to limit the lidocaine dose for local anesthesia in cats to no more than 3–4 mg/kg [9,10,11,12].

Locoregional anesthesia for feline castration can be achieved by intratesticular, sacrococcygeal, and epidural block [13,14]. Intratesticular block is the recommended technique for castration in both dogs and cats as the local anesthetic travels to the spermatic cord and alleviates pain during spermatic cord ligation. Although intratesticular block is effective and should be performed, its use may be limited due to technical challenges, skill level, knowledge, or complications. It should be avoided in cases of testicular tumors or infections. Complications of intratesticular block include tissue injury, bruising at the injection site, and risk of systemic absorption [13,15]. Therefore, an alternative local anesthetic technique for feline castration is warranted.

Instillation is a common technique used in local anesthesia such as intraperitoneal and incisional block. This technique is simple and safe as it does not require special skills or techniques [11,16,17]. However, studies on spermatic cord instillation in feline castration are limited. Therefore, the objective of this study was to compare the perioperative analgesic efficacy of lidocaine intratesticular block and spermatic cord instillation in feline castration. The hypothesis of this study was that lidocaine spermatic cord instillation would provide intraoperative analgesic efficacy similar to that of intratesticular block and that both techniques would offer superior intraoperative analgesic effects compared to the control group.

## 2. Materials and Methods

The study was approved by the Institutional Animal Care and Use Committee of Khon Kaen University, Thailand (IACUC-KKU-27/66). All owners were informed and gave their written consent prior to inclusion.

### 2.1. Cats

Forty-five client-owned male cats of various breeds, aged between 6 months and 5 years and weighing between 2 and 8 kg, were included in this study. The cats were randomly and equally divided into three groups: intratesticular block (IT), spermatic cord instillation (S), and control (C) groups (*n* = 15 cats/group). All cats were determined as healthy and classified according to the American Society of Anesthesiologists risk (ASA) class I based on physical examinations and blood profiles, including complete blood count, creatinine, aspartate transaminase, total protein, and albumin. Food was withheld from the cats for 4–6 h prior to surgery. Cats with cryptorchism or reproductive diseases were excluded from the study.

This study was a prospective clinical trial conducted from May 2023 to July 2024 at the Veterinary Teaching Hospital, Faculty of Veterinary Medicine, Khon Kaen University, Thailand.

### 2.2. Anesthesia and Castration

Before surgery, cats were physically examined to establish baseline parameters (T0) including heart rate (HR), respiratory rate (RR), systolic blood pressure (SBP), mean blood pressure (MBP), and diastolic blood pressure (DBP). All cats were premedicated with intramuscular acepromazine at 0.02 mg/kg (Combistress, Kele N.V., Hoogstraten, Belgium). After 10–15 min, lactated ringer’s solution (Lactated ringer’s, General Hospital Products Public CO., Pathum Thani, Thailand) was administered intravenously via the cephalic vein at a rate of 3 mL/kg/h. Cefazolin at 25 mg/kg, (Cefaben^®^, L.B.S. Laboratory LTD., Bangkok, Thailand) was intravenously administered as antibiotic prophylaxis. Cats were anesthetic induced with propofol (Profol^TM^ 1%, Baxter Pharmaceuticals India Private Limited, Gujarat, India), intravenously titrated to effect until endotracheal intubation was achieved (6.8 ± 1.5 mg/kg, range: 4.33–10.81 mg/kg). Anesthesia was maintained with isoflurane inhalation (Attane^TM^, Piramal Critical Care, Inc., Bethlehem, PA, USA) using a non-rebreathing system with an oxygen flow rate of 200–400 mL/kg/min. The isoflurane concentration was maintained at 1.5–2% and adjusted as necessary based on significant changes in intraoperative vital signs. Before surgery, fentanyl at 1 µg/kg (Fentanyl-Hameln 50 µg/mL, Siam Bioscience Co., Nonthaburi, Thailand) was administered intravenously as preventive analgesia.

Intraoperative parameters, including HR, RR, oxygen saturation (SpO_2_), end-tidal carbon dioxide (ETCO_2_), isoflurane concentration, and minimum alveolar concentration (MAC) of isoflurane, were recorded using multiparametric monitoring (BM7 VET Pro, Bionet Co., Ltd., Gunpo, Republic of Korea). SBP, MBP, and DBP were measured using an oscillometer (BP-Accuguard^TM^, Vmed Technology, Mill Creek, WA, USA). These parameters were recorded at the following timepoints: T1 (before incision), T2 (during extraction of the left testis), T3 (after removal of the left testis), T4 (during extraction of the right testis), and T5 (after removal of the right testis). In the IT group, lidocaine was injected into the testes, and NSS was instilled onto the spermatic cord. In the S group, NSS was used for intratesticular block and lidocaine was applied for spermatic cord instillation. In the C group, NSS was used in both intratesticular block and spermatic cord instillation. Lidocaine (Lidocaine hydrochloride 2% *w/v*, The Government Pharmaceutical Organization, Bangkok, Thailand) at 2 mg/kg was diluted with 0.9% saline solution (NSS, General Hospital Products Public CO., Pathum Thani, Thailand) to obtain a volume of 0.3 mL per testis, and the same volume of NSS was also prepared. The intratesticular block was performed by injecting the solution into the testis using a 30-gauge, half-inch needle attached to a 1 mL syringe (Nipro, Shanghai Kindly Enterprise Development Group Co., Ltd., Shanghai, China), with the needle directed toward the spermatic cord [5], and surgery began two minutes after the administration. For spermatic cord instillation, following the incision and extraction of each spermatic cord, the solution was instilled (0.3 mL per testis) onto the spermatic cord within the scrotal sac until fully administered. Leakage of the solution was prevented for at least two minutes, followed by ligation and removal of the testis. Closed castration using the overhand technique was performed in all cats. After the removal of both testes, isoflurane inhalation was discontinued and extubation was performed when a strong palpebral reflex was noticed.

After surgery, meloxicam (Metacam, Boehringer Ingelheim, Ingelheim/Rhein, Germany) at 0.2 mg/kg was subcutaneously administered immediately after surgery. All cats were anesthetized by the same anesthetist (T.M.) and closed castration was performed by the same experienced surgeon (T.S.), both of whom were blinded to the group assignments.

### 2.3. Postoperative Pain Assessment

Pain was scored using CMPS-feline and CSU-feline at T6 (60 min after extubation) and T7 (120 min after extubation). For cats with a CMPS-feline score of 5/20, morphine (Morphine Sulfate Injection, M & H Manufacturing, Samut Prakan, Thailand) at 0.2 mg/kg was administered intramuscularly as rescue analgesia. Pain assessment was performed by the same examiner (T.M.) who was blinded to the group assignments. Vital signs, including HR, RR, SBP, MBP, and DBP, were recorded at T6 and T7.

### 2.4. Statistical Analysis

The sample size was calculated using a formula for comparing two independent means based on MBP data at T3 from a pilot study (MBP in the S group was 78.3 ± 9.3 and in the C group, it was 102.0 ± 30.0), with a specified power of 0.8 and a type I error probability of 0.05, respectively. According to the calculation, the sample size required 14 cats per group. All data were tested for normality using the Shapiro–Wilk test. Preoperative data including age, body weight, HR, RR, SBP, MBP, DBP, and pain scores were compared between groups using one-way analysis of variance (ANOVA) for parametric parameters and the Kruskal–Wallis test for nonparametric parameters. Intraoperative data including HR, RR, SBP, MBP, DBP, SpO_2_, ETCO_2_, isoflurane concentration and MAC at T1–T5 were analyzed using a linear mixed model with repeated measurements. Postoperative data, including HR, RR, SBP, MBP, DBP, CMPS-feline, and CSU-feline pain scores were compared using a linear mixed model with repeated measurements. Treatment groups, timepoints, and their interaction were used as fixed factors, while the random factor was individual cats. The Bonferroni adjustment was applied for pairwise comparison between groups at each timepoint and across the timepoints for each group. All statistical analyses were performed using the STATA software (STATA v18, University licensed, StataCorp LLC, College Station, TX, USA), and *p* < 0.05 was determined as statistically significant.

## 3. Results

Forty-five male cats (15 cats/group) were included in the study. In the demographic data, no significant differences were found in age, body weight, HR, RR, SBP, MBP, DBP, surgical duration, and anesthetic duration between groups (Table 1). During castration, bruising of the testes was observed in five cats. However, no complications related to anesthesia or surgery were noted.

The intraoperative parameters including HR, RR, SpO_2_, ETCO_2_, and isoflurane concentration did not significantly differ between groups (Table 2). Noninvasive blood pressure measurements showed that SBP, MBP, and DBP were significantly lower in the S group compared to the C group at T3–T5 (*p* < 0.01). MBP and DBP were significantly lower in the IT group compared to group C at T3–T4. No significant differences in any intraoperative parameters were observed between the IT and S groups. The isoflurane MAC values in the C group were higher than those in the IT and S group, but the differences were not statistically significant.

RR, SpO_2_, isoflurane concentration, and MAC values did not significantly differ between intraoperative timepoints. In the IT group, HR was significantly increased at T2 compared to the other timepoints. In the S group, HR was significantly increased at T2 compared to T3 and T5 and at T4 compared to T5 (*p* < 0.0003). In the IT group, SBP, MBP, and DBP did not significantly change between intraoperative timepoints. In the S group, SBP, MBP, and DBP significantly increased at T2 compared to T3 (*p* < 0.005). In the C group, SBP significantly increased at T4 compared to T1–T2, and MBP and DBP significantly increased at T3–T5 compared to T2 (*p* < 0.0002). In the IT group, ETCO_2_ at T2 significantly increased compared to T1 (*p* < 0.0009) (Table 2).

Postoperative parameters, HR, RR, SBP, MBP, and DBP, were not significantly different between groups (Table 3). In the IT group, HR decreased significantly at T7 compared to T6 (*p* < 0.03). Pain scores assessed using CMPS-feline and CSU-feline in the IT and S group were lower than in the C group, but the differences were not significant. In the S and C group, CMPS-feline pain scores significantly decreased at T7 compared to T6 (*p* < 0.001). Morphine was administered as rescue analgesia in two cats in the IT group, four cats in the S group, and four cats in the C group.

## 4. Discussion

Local anesthesia can effectively reduce intraoperative noxious stimulation by completely inhibiting the transmission of nociceptive impulses [11], thereby minimizing the dosages of inhalant anesthesia and mitigating the side effects of general anesthesia in most surgeries including feline castration [11,13,15]. In this study, noninvasive blood pressure was lower in the IT and S groups compared to the C group. No intraoperative parameters were significantly different between the IT and S groups. Consistent with previous studies, these findings indicate that both lidocaine intratesticular block and spermatic cord instillation can reduce noxious stimulation during feline castration [13,15]. Therefore, spermatic cord instillation can serve as a local anesthetic option for feline castration.

Intratesticular block is the recommended local anesthetic technique for castration [13,15,18,19,20]. It is a simple procedure that provides an analgesic effect before incision, aligning with preventive analgesic protocol. However, its limitations include unsuitability for cases involving testicular tumors or infections and the potential for bruising at the injection site. In contrast, spermatic cord instillation is a simple local anesthetic technique without the risk of testicular bruising. However, this technique can only be performed after incision, when noxious stimulation has already occurred. In this study, noninvasive blood pressure in the IT group did not significantly change between intraoperative timepoints demonstrating the efficacy of intratesticular block in inhibiting noxious stimulation throughout the castration period. On the other hand, in the S group, noninvasive blood pressure during extraction was significantly higher than after spermatic cord instillation and testicular removal. Additionally, HR significantly increased at the time of extraction of each testis compared to at the time of testicular removal. Given the 2 min onset time of lidocaine [9], intratesticular block does not delay surgical time, whereas spermatic cord instillation requires lidocaine contact time, which may slightly prolong the procedure. The results of this study suggest that intratesticular block is superior to spermatic cord instillation in terms of ease of technique handling, controlling intraoperative noxious stimulation, and requiring fewer rescue analgesia. The choice of local block technique should be based on the advantages, disadvantages, and limitations of each technique. Therefore, intratesticular block remains the recommended technique, while spermatic cord instillation can be used as an optional or additional approach for local anesthetic block in feline castration.

Noxious stimuli during surgery trigger activated nociceptors, even under general anesthesia [8]. Blood pressure and heart rate often fluctuate over time due to regulatory mechanisms that maintain cardiovascular homeostasis [21]. Blood pressure and heart rate have been commonly used in several studies for monitoring intraoperative nociceptive responses and to measure the response to local anesthesia during neutering procedures [13,15,16,17,19,20,22,23,24]. However, these measurements can be influenced by various factors such as stress, medications, and health conditions, and further measurements are required to assess intraoperative noxious stimulation. In human medicine, alternative tools such as heart rate variability (HRV), analgesia nociception index (ANI), and the surgical pleth index (SPI) are used to predict nociception under general anesthesia [25,26,27,28]. Although there is currently no gold standard for evaluating intraoperative nociceptive responses, further studies in veterinary medicine are warranted to investigate the usefulness of these instruments in assessing noxious stimulation during the perioperative period in association with intraoperative vital signs.

Appropriate pain management can reduce pain scores and the need for analgesics after surgery. Multimodal analgesia, which includes preoperative opioids, local anesthesia, and NSAIDs, is effective in minimizing perioperative pain [5,7]. In this study, although pain scores were low in all cats due to the use of fentanyl and meloxicam, pain scores in the C group were higher than in the IT and S group and more cats required rescue analgesia. Moreover, intraoperative vital signs (SBP, MBP, DBP) in the C group were significantly higher than in the IT and S groups, and vital signs (HR, SBP, MBP, DBP) significantly increased at the time of extraction and removal of the testes. Therefore, the use of fentanyl and meloxicam without local block was inadequate for controlling pain during castration. Based on the intraoperative findings, we can preliminarily conclude that both intratesticular block and spermatic cord instillation can be used as local anesthetic techniques for feline castration. However, considering both intraoperative and postoperative outcomes, intratesticular block appears to be the most effective method for providing analgesia in feline castration. Even though local anesthesia was applied in the IT and S groups, some cats exhibited high pain scores, and rescue analgesia was still needed, suggesting that additional pain management is required for controlling pain during castration. Further studies on pain management strategies, in addition to pre-emptive opioids, local block with intratesticular block and spermatic cord instillation, and perioperative NSAIDs are necessary to improve the quality of pain management in feline castration.

This study has some limitations. The lack of significant differences in intraoperative and postoperative parameters between intratesticular block and spermatic cord instillation may be attributed to the multimodal analgesic used and the small sample size. Additionally, feline castration causes mild pain intensity, and the use of fentanyl as preventive analgesia may have influenced the measured variables and noxious stimulation, resulting in nonsignificant findings. Another limitation was the adjustment of isoflurane concentration. The isoflurane MAC in the IT and S groups was lower than in the controls but without statistical significance as isoflurane concentration was consistently maintained at 1.5–2% unless vital signs showed notable changes. Adjusting isoflurane concentration based on the depth of the anesthesia is recommended to better evaluate its effects on isoflurane MAC. Moreover, the end-tidal isoflurane concentration should be used, as it provides real-time feedback for MAC measurement and facilitates target-controlled titration of inhaled anesthetics [29], ensuring more accurate real-time measurements. In this study, postoperative pain scores were assessed up to 120 min after extubation, but prolonging the postoperative recovery time may be warranted. Further research is required to improve the quality of perioperative pain management in feline castration.

## 5. Conclusions

In this study, both intratesticular and spermatic cord instillation of lidocaine were proven to reduce intraoperative blood pressure, indicating their efficacy in inhibiting noxious stimuli during feline castration. Intratesticular block is recommended as it provides preventive analgesia and leads to more stable intraoperative vital signs and lower postoperative pain scores. Therefore, lidocaine spermatic cord instillation can be considered as an optional local anesthetic technique for pain management in feline castration.

## Figures and Tables

**Table 1:** Demographic data in intratesticular block (IT), spermatic cord instillation (S), and control (C) groups.

Parameter	Group		
	IT	S	C
Age (months)	14.7 ± 9.6	11.4 ± 5.1	12.7 ± 7.7
Body weight (kg)	4.3 ± 0.9	4.0 ± 0.5	4.2 ± 0.8
Heart rate (bpm)	205.4 ± 23.3	203.7 ± 27.8	210.3 ± 24.8
Respiratory rate (bpm)	75.1 ± 28.5	79.2 ± 28.9	61.2 ± 17.6
SBP (mmHg)	144.5 ± 18.6	131.5 ± 20.4	140.6 ± 27.6
MBP (mmHg)	122.0 ± 16.1	107.2 ± 17.2	111.0 ± 23.1
DBP (mmHg)	110.5 ± 16.8	95.2 ± 18.3	96.7 ± 24.5
Anesthetic duration (min)	27.2 ± 5.7	28.8 ± 5.3	25.5 ± 3.0
Surgical duration (min)	9.2 ± 1.4	9.5 ± 1.9	9.0 ± 1.3

Data were represented as mean ± standard deviation. IT, intratesticular block; S, spermatic cord instillation; C, control. SBP, systolic blood pressure; MBP, mean blood pressure; DBP, diastolic blood pressure; bpm, beats per minute.

**Table 2:** Intraoperative parameters in the intratesticular block (IT), spermatic cord instillation (S), and control (C) groups.

Parameter	Group	Timepoint				
		T1	T2	T3	T4	T5
HR (bpm)	IT	143.1 ± 26.5 ^a^	160.0 ± 25.1 ^b^	144.6 ± 28.9 ^a^	151.3 ± 29.0 ^a^	148.9 ± 28.5 ^a^
	S	154.9 ± 36.5 ^abc^	165.8 ± 25.1 ^b^	147.0 ± 21.2 ^ac^	157.8 ± 31.0 ^ab^	141.6 ± 15.2 ^c^
	C	147.4 ± 30.6	155.9 ± 26.3	156.1 ± 26.4	141.5 ± 29.8	152.8 ± 27.8
RR (bpm)	IT	27.0 ± 4.9	25.1 ± 4.7	24.1 ± 7.1	26.1 ± 9.7	23.6 ± 6.3
	S	28.7 ± 10.7	25.4 ± 7.1	25.8 ± 8.0	25.7 ± 9.1	26.4 ± 8.0
	C	23.3 ± 10.0	20.5 ± 9.1	21.9 ± 7.6	23.5 ± 12.3	22.0 ± 8.9
SBP (mmHg)	IT	117.1 ± 20.6	124.4 ± 29.5	120.8 ± 25.7 ^AB^	129.6 ± 27.5 ^AB^	127.7 ± 27.9 ^AB^
	S	121.1 ± 30.2 ^ab^	123.0 ± 20.5 ^a^	107.7 ± 11.6 ^b,B^	120.6 ± 15.3 ^ab,B^	109.1 ± 12.0 ^ab,B^
	C	124.5 ± 23.6 ^a^	124.8 ± 17.5 ^a^	138.7 ± 24.3 ^ab,A^	145.7 ± 24.9 ^b,A^	135.9 ± 20.9 ^ab,A^
MBP (mmHg)	IT	86.3 ± 25.2	88.0 ± 22.0	81.9 ± 16.5 ^B^	89.1 ± 20.9 ^B^	88.8 ± 26.6 ^AB^
	S	89.5 ± 29.3 ^a^	90.5 ± 18.9 ^a^	74.3 ± 9.5 ^b,B^	86.3 ± 11.6 ^ab,B^	76.3 ± 11.8 ^ab,B^
	C	87.5 ± 17.9 ^ac^	84.8 ± 15.7 ^a^	102.7 ± 22.2 ^bc,A^	102.5 ± 19.6 ^bc,A^	102.5 ± 19.6 ^bc,A^
DBP (mmHg)	IT	71.1 ± 25.9	69.9 ± 19.3	62.4 ± 16.2 ^B^	68.7 ± 19.3 ^B^	72.8 ± 28.3 ^A,B^
	S	75.4 ± 32.5 ^a^	73.3 ± 17.8 ^a^	57.2 ± 11.6 ^b,B^	68.2 ± 13.1 ^ab,B^	59.9 ± 13.5 ^ab,B^
	C	69.9 ± 18.6 ^ac^	65.1 ± 17.4 ^a^	85.4 ± 23.2 ^bc,A^	92.5 ± 24.4 ^b,A^	86.1 ± 21.2 ^bc,A^
SpO_2_ (%)	IT	98.0 ± 1.4	97.4 ± 1.5	97.6 ± 1.2	97.8 ± 1.3	97.9 ± 1.1
	S	97.3 ± 1.8	96.9 ± 2.0	97.2 ± 1.7	97.1 ± 2.2	97.7 ± 1.3
	C	97.8 ± 1.3	97.6 ± 1.6	97.7 ± 1.0	97.1 ± 1.4	97.5 ± 1.7
ETCO_2_ (%)	IT	28.1 ± 4.5 ^a^	31.3 ± 5.5 ^b^	30.4 ± 6.1 ^ab^	29.9 ± 6.8 ^ab^	29.2 ± 4.6 ^ab^
	S	29.3 ± 3.9	29.8 ± 4.7	29.8 ± 4.1	30.2 ± 4.8	28.6 ± 4.5
	C	30.8 ± 6.9	31.2 ± 6.7	31.2 ± 6.3	30.5 ± 6.1	29.8 ± 6.5
ISO (%)	IT	1.9 ± 0.2	1.9 ± 0.4	1.8 ± 0.3	1.8 ± 0.3	1.7 ± 0.3
	S	1.9 ± 0.2	2.0 ± 0.9	1.9 ± 0.4	1.8 ± 0.5	1.8 ± 0.5
	C	1.9 ± 0.2	2.0 ± 0.4	1.9 ± 0.2	1.9 ± 0.4	1.8 ± 0.2
MAC (%)	IT	1.2 ± 0.2	1.2 ± 0.2	1.2 ± 0.2	1.2 ± 0.2	1.1 ± 0.3
	S	1.2 ± 0.2	1.2 ± 0.2	1.2 ± 0.3	1.3 ± 0.4	1.2 ± 0.3
	C	1.3 ± 0.3	1.4 ± 0.3	1.3 ± 0.2	1.3 ± 0.2	1.3 ± 0.2

Data were represented as mean ± standard deviation. ^a–c^ Different superscript small letters indicate significant differences within groups between timepoints (*p* < 0.01). ^A–C^ Different superscript capital letters indicate significant differences between groups (*p* < 0.01). IT, intratesticular block; S, spermatic cord instillation; C, control; HR, heart rate; RR, respiratory rate; SBP, systolic blood pressure; MBP, mean blood pressure; DBP, diastolic blood pressure; SpO_2_, oxygen saturation; ETCO_2_, end-tidal carbon dioxide; and MAC, minimum alveolar concentration. ISO, isoflurane concentration. T1, before incision; T2, during extraction of the left testis; T3, after removal of the left testis; T4, during extraction of the right testis; T5, after removal of the right testis.

**Table 3:** Postoperative parameters in the intratesticular block (IT), spermatic cord instillation (S), and control (C) groups.

Parameter	Group	Timepoint	
		T6	T7
HR (bpm)	IT	204.0 ± 31.2 ^a^	187.5 ± 28.5 ^b^
	S	199.6 ± 24.5	194.8 ± 22.9
	C	205.7 ± 14.9	201.7 ± 20.9
RR (bpm)	IT	42.9 ± 20.2	46.1 ± 12.8
	S	44.3 ± 21.7	52.3 ± 23.3
	C	42.4 ± 11.6	47.2 ± 16.6
SBP (mmHg)	IT	143.7 ± 36.4	140.2 ± 26.8
	S	138.6 ± 22.9	138.9 ± 23.0
	C	144.9 ± 21.1	140.0 ± 29.5
MBP (mmHg)	IT	108.3 ± 18.1	113.1 ± 18.2
	S	119.8 ± 19.5	117.2 ± 22.7
	C	122.1 ± 19.7	121.7 ± 24.6
DBP (mmHg)	IT	97.5 ± 24.5	100.7 ± 17.6
	S	108.7 ± 20.1	106.7 ± 24.8
	C	110.7 ± 20.2	112.3 ± 23.0
CMPS-feline	IT	3.0 ± 1.7	2.8 ± 1.6
	S	4.1 ± 3.0 ^a^	2.0 ± 1.5 ^b^
	C	4.6 ± 1.7 ^a^	3.2 ± 1.6 ^b^
CSU-feline	IT	0.8 ± 0.4	0.9 ± 0.6
	S	0.9 ± 0.6	0.6 ± 0.7
	C	1.1 ± 0.8	0.9 ± 0.6

Data were represented as mean ± standard deviation. ^a,b^ Different superscript small letters show significant differences within groups between timepoints *(p* < 0.01). IT, intratesticular block; S, spermatic cord instillation; C, control. HR, heart rate; RR, respiratory rate; SBP, systolic blood pressure; MBP, mean blood pressure; DBP, diastolic blood pressure; CMPS-feline, Glasgow composite measure pain scale-feline; CSU-feline, Colorado acute pain scale; T6, 60 min after extubation; T7, 120 min after extubation.

## Data Availability

All data are contained within this article.
